# Implementing Green Analytical Methodologies Using Solid-Phase Microextraction: A Review

**DOI:** 10.3390/molecules25225297

**Published:** 2020-11-13

**Authors:** Kayla M. Billiard, Amanda R. Dershem, Emanuela Gionfriddo

**Affiliations:** 1 Department of Chemistry and Biochemistry, College of Natural Sciences and Mathematics, The University of Toledo, Toledo, OH 43606, USA; Kayla.Billiard@rockets.utoledo.edu; 2 Department of Chemistry, College of Arts and Sciences, Siena Heights University, Adrian, MI 49221, USA; dershem.amanda@gmail.com; 3 Dr. Nina McClelland Laboratory for Water Chemistry and Environmental Analysis, The University of Toledo, Toledo, OH 43606, USA; 4 School of Green Chemistry and Engineering, The University of Toledo, Toledo, OH 43606, USA

**Keywords:** solid-phase microextraction, green analytical chemistry, sample preparation, greenness assessment, Green Analytical Procedure Index

## Abstract

Implementing green analytical methodologies has been one of the main objectives of the analytical chemistry community for the past two decades. Sample preparation and extraction procedures are two parts of analytical method development that can be best adapted to meet the principles of green analytical chemistry. The goal of transitioning to green analytical chemistry is to establish new methods that perform comparably—or superiorly—to traditional methods. The use of assessment tools to provide an objective and concise evaluation of the analytical methods’ adherence to the principles of green analytical chemistry is critical to achieving this goal. In this review, we describe various sample preparation and extraction methods that can be used to increase the greenness of a given analytical method. We gave special emphasis to modern microextraction technologies and their important contributions to the development of new green analytical methods. Several manuscripts in which the greenness of a solid-phase microextraction (SPME) technique was compared to other sample preparation strategies using the Green Analytical Procedure Index (GAPI), a green assessment tool, were reviewed.

## 1. Introduction

### 1.1. Green Analytical Chemistry

One of the growing trends in the chemistry community is the implementation of green chemistry and the emphasis on green chemistry in the lab. Chemists worldwide are attempting to replace traditional methods and reactions with more sustainable, energy-efficient alternatives. Green chemistry can take many forms, from re-designing experiments with more sustainable materials to active waste management [1]. Finding a balance between cost-effective techniques and sustainable materials can be difficult, particularly when evaluating the trade-offs between alternative technologies [2]. For example, replacing a reagent in a reaction may reduce waste production, but it may also result in more toxic waste products. To understand the total greenness of a particular analytical method, all facets of the method must be understood [3]. Several systems have been developed to evaluate the effectiveness of green chemistry techniques compared to traditional techniques [4,5,6,7,8]. These systems are based on the 12 Principles of Green Chemistry and were redefined as the 12 Principles of Green Analytical Chemistry as outlined in Table 1 [1,5].

Green chemistry principles are now widely being incorporated into academic programs [9,10] as a means to train new scientists to consider the environmental impacts of their research. Many undergraduate and graduate programs now include curricula that apply green chemistry principles to older, more widely used chemistry techniques. Likewise, many research groups are seeking to revolutionize their testing methods to fit within the green chemistry framework.

Analytical chemistry is one such field that is attempting to apply green chemistry principles to instrumentation and analytical techniques. In analytical chemistry, one of the largest challenges is developing more sustainable and efficient sample preparation techniques [3,7,11,12]. Green sample preparation is the most accessible application of green analytical chemistry; it does not require the overhaul of well-established instrumental methods, yet it still provides a tangible green impact on lab practices. All analytical chemists must use some form of sample preparation, and vast quantities of samples are analyzed in labs worldwide every day. Applying green chemistry principles to sample preparation leads to substantial socioeconomic and environmental impacts. As evidenced by the 12 Principles of Green Analytical Chemistry [5,13] (Table 1), many of the opportunities for increasing the sustainability and greenness of an analytical lab are directly related to the sample preparation process.

As the first principle outlines, the most efficient way to reduce waste is to introduce the sample with little to no preparation. Unfortunately, most analytes must first be extracted from their matrices before analysis. In the cases where sample preparation is necessary, the second, fifth, seventh, ninth, tenth, eleventh, and twelfth principles can and should be applied. Sample preparation techniques should be optimized to reduce energy input, solvent use, waste production, and operator exposure as much as possible. This can be accomplished in a variety of ways, including the restriction of the size and number of samples, the use of reusable sample extraction devices [14], and avoiding toxic organic solvents.

To evaluate a specific analytical method for its greenness, the method must be considered using the 12 Principles of Green Analytical Chemistry as highlighted above. Specifically, each method must be broken down into the required steps necessary for carrying out the experiment and each step must be analyzed for potential issues. Some metrics have been established to aid in the process of evaluating greenness, including the Green Analytical Procedure Index (GAPI) [8].

### 1.2. Overview of Sample Preparation Strategies and Their Impact on Analytical Methods Greenness

A myriad of sample preparation strategies exist as reliable, go-to methods for analytical chemists. Throughout the field’s history, these traditional methods have evolved to become increasingly efficient and precise. Despite their ubiquity, some of these methods—such as liquid–liquid extraction (LLE) and Soxhlet extraction—are being reconsidered because they do not align with the principles of Green Analytical Chemistry (GAC). The preparation and extraction portion of an analytical procedure is often the most polluting component [11]; as such, it is often the focus of laboratories and industries that aim to make their methods greener. However, given the importance of sample preparation, the quality of analysis should not be unnecessarily compromised in pursuit of greener methods. In other words, a good method to replace those of the past should be greener and also provide equal or better analytical performance.

Early attempts at greener methods utilized various forms of heat, pressure, or radiation to facilitate the extraction process. These methods—including ultrasound-assisted extraction (UAE), pressurized solvent extraction (PSE), microwave-assisted extraction (MAE), and supercritical fluid extraction (SFE)—successfully improved the greenness of extraction compared to their predecessors [6]. UAE involves exposing the sample to ultrasound waves which agitate the molecules and expedite the extraction process. While this method increases the efficiency of extraction, it still requires a high volume of solvent. Similarly, PSE works by using solvents that are near their supercritical region. The high temperatures allow for more efficient extraction. However, similar to UAE, this method still requires a high volume of solvent. Additionally, the use of solvents at such high temperatures also demands a great deal of energy. 

MAE uses microwaves to quickly heat the solvent and the sample in a closed container. This method provides a more efficient extraction and reduces the volume of solvents required. Furthermore, the temperature and pressure conditions for MAE allow water to be used as the extracting solvent rather than an organic solvent. Eliminating the necessity of an organic solvent is more environmentally friendly and is also safer for the user [15]. As with UAE and PSE, the greener aspects of this method come at the price of greater energy consumption. SFE facilitates extraction by directing the flow of supercritical CO_2_ through the sample and using it as the extraction fluid. In this context, supercritical CO_2_ is considered a green solvent because it is non-toxic, non-flammable, abundant, renewable, and does not produce waste [16]. Maintaining supercritical CO_2_ causes this method to require energy, similar to the extraction methods listed above.

Another method of sample preparation known as Quick Easy Cheap Effective Rugged Safe (QueEChERS) works by extracting analytes using an organic solvent, partitioning of two phases by the addition of a salt, and purifying the supernatant by dispersive solid-phase extraction (dSPE) [17]. This method can extract hundreds of different analytes, making it particularly useful for complex samples. Although the QuEChERS method is useful, its use of organic solvents and high production of laboratory waste are not in accordance with GAC principles. In an attempt to improve the greenness of this method, miniaturized QuEChERS kits were developed [18]. As the name suggests, these kits perform the same analysis but on a smaller scale. While the miniature QuEChERS kits lessen the amount of laboratory waste produced as well as the amount of sample required in the first place, efforts are being made to develop extraction methods with even lower environmental impact. 

A recent development in the field of green analytical chemistry known as microextraction has taken great steps forward in achieving that goal. Microextraction, which is not to be confused with mere miniaturization, is a nonexhaustive technique where the extraction phase volume is very small [14]. This means that the materials and solvents required for extraction are greatly minimized, as is the amount of sample that is required for analysis. One particular type of microextraction, known as solid-phase microextraction (SPME), utilizes solid material for the extraction phase. The green aspects of microextraction combined with the green components of solid-phase extraction result in a technique that is quick, uses little to no solvent, requires little to no sample preparation, and produces very little laboratory waste [19]. The aim of this review is to evaluate and compare the greenness of various sample preparation methods using the Green Analytical Procedure Index (GAPI). Table 2 provides an overview of the most commonly used sample preparation and extraction procedures considering their relative greenness factors.

### 1.3. Solid-Phase Microextraction

#### 1.3.1. Principles

Solid-phase microextraction is a technique that utilizes an extraction phase coated on a solid support [13]. This thinly coated layer (7–250 µm in thickness) serves as the extraction phase where analytes diffuse from the sample matrix to the coating by partitioning into the bulk of the coating or onto its porous active surface until equilibrium is reached. The amount of analyte that is able to be extracted by this method is dependent upon the equilibrium between the sample matrix and the extraction phase. The extracted amount at equilibrium is given by the equation

$$n^{eq}=\frac{K_{es}V_{e}V_{s}}{K_{es}V_{e}+V_{s}}C_{s\;},$$

where *K_es_* is the distribution coefficient of the analyte between the matrix and extraction phase, *V_e_* is the volume of the extraction phase, *V_s_* is the volume of the sample, and *C_s_* is the initial concentration of the analyte in the sample. However, in a situation where the volume of the sample is substantially greater than the sum of the distribution coefficient and the volume of the extraction phase, the equation can be simplified to

$$n^{eq}=K_{es}V_{e}C_{s\;}.$$

This simplification allows for precise quantification and extraction optimization even in situations where the volume of the sample is unknown. For example, analyzing the components of river water could be done quantitatively, even though the volume of the water in the river could never reliably be known. It should be noted that solid-phase microextraction is a non-exhaustive extraction method, and it is dependent on partitioning equilibria [14]. Despite this, the amount of analyte extracted is directly proportional to the original concentration of an analyte in the sample, as seen in the equation above. As such, a calibration curve can be created for targeted analytes, thus allowing for quantitative analysis. Moreover, SPME enables performing extraction and preconcentration simultaneously, further reducing the number of analytical steps needed to achieve desired method sensitivity [14].

With respect to optimization, it can be seen that extraction is enhanced when the distribution coefficient, *K_es_*, and the kinetics of the extraction can be hastened by optimizing the volume of the extraction phase, *V_e_*, and its thickness [14]. The distribution coefficient is optimized primarily by choosing the best extraction phase coating by considering the chemical properties of the analytes and, if applicable, the sample matrix. Many configurations of SPME inherently optimize the volume of the extraction phase to enhance the high surface-area-to-volume ratio of the extraction phase coating [20]. 

#### 1.3.2. Configurations

Of particular interest in SPME is the geometry—or configuration—of the extraction device. The available surface area of the extraction phase is important in determining the efficiency and kinetics of the extraction. In particular, thin-film-coated substrates of any geometry can affect the sample size or concentration requirements—resulting enhanced extraction efficiency [20,21].

**Figure 1:**
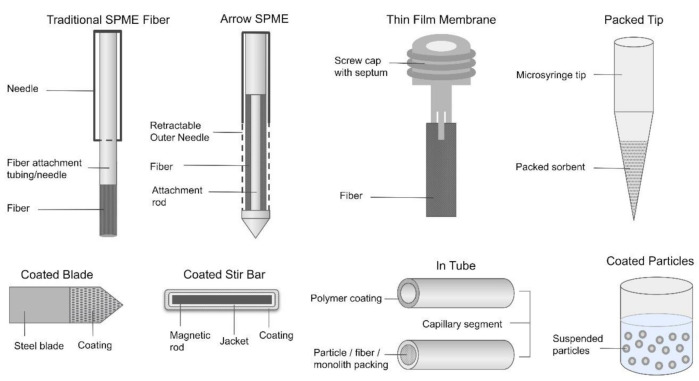
Solid-phase microextraction geometries. Figures not to scale.

Many geometries exist for SPME (Figure 1), the main categories of which are planar, spherical, rod, and in-tube or cylinder [22]. Arrow geometries, a subcategory in which a rod or planar geometry tapers to a point, are also commonplace [23]. The geometry chosen for analysis depends on the constraints of the analyte and its matrix. Generally, the smaller the diameter or length of the extraction phase, the higher its surface-area-to-volume ratio will be, which results in a shorter extraction period and a higher amount of analytes extracted [20].

The first developed SPME device was in the fiber configuration [24]. The fiber geometry has a low surface area that results in higher detection limits and relatively slower equilibration times. Planar and in-tube geometries were developed to provide an alternative to the fiber geometry with an increased surface area/volume ratio able to facilitate more efficient extraction [23].

Thin-film solid-phase microextraction (TF-SPME) was developed as a strategy for increasing extraction efficiency, ensuring fast extraction kinetics [25]. Any SPME geometry can be turned into a thin-film geometry by way of coating a traditional geometry with an extraction phase. There are many methods for coating, the most common of which are dipping the support into a slurry of the extraction phase, spreading the extraction phase on the support (also sometimes referred to as “bar coating”), spraying the extraction phase, and spinning/electrospinning [20,26].

Thin-film SPME can be used for an array of applications such as bioanalysis, food, and environmental monitoring. For example, SPME is often used to analyze air quality or water quality in environmental studies, the presence of specific nutrients or toxins in food, and the existence of specific compounds in pharmaceuticals—or of pharmaceuticals in other matrices [27,28,29,30,31]. Frequently, TF-SPME methods can replace existing methods for analyzing these compounds; as such it is necessary to compare the existing methods with the newly developed TF-SPME methods to ascertain that the new methods are indeed more sustainable—and that the analysis results are of comparable or superior quality [27,32].

SPME methods provide an improvement in the greenness of analytical methods by reducing the waste, labor costs, and resources necessary to complete the procedure. Besides the general improvements related to greenness aspects, SPME often offers unique opportunities for in vivo sampling and constitutes a valid alternative to more labor-intensive methods.

To elucidate just how effective SPME can be compared to other methods, examples of analytical methods were collected and evaluated using the Green Analytical Procedure Index (GAPI) assessment. Traditional methods were compared to methods that utilize SPME for analysis and pictograms were constructed to summarize those findings.

## 2. Introduction to the GAPI Method for Analytical Methods Greenness Evaluation

The GAPI is a greenness evaluation method that can be used to determine how well an analytical procedure adheres to the principles of GAC. This evaluation considers a method in its entirety and takes into account five major categories: general method type, sample collection, sample preparation, reagents and solvents required, and instrumentation [8]. Within each of these categories, specific criteria are evaluated on whether their adherence to the principles of GAC are high, medium, or low. For example, within the category of sample preparation, the extraction is evaluated as being nanoscale, microscale, or macroscale. Within the reagents and solvents category, reagent health and safety hazards are evaluated for having hazard ratings of 0–1, 2–3, or 4. There are a total of 15 criteria that are used in the GAPI greenness evaluation tool, each containing three tiers of GAC adherence.

### Description of Pictograms

**Figure 2:**
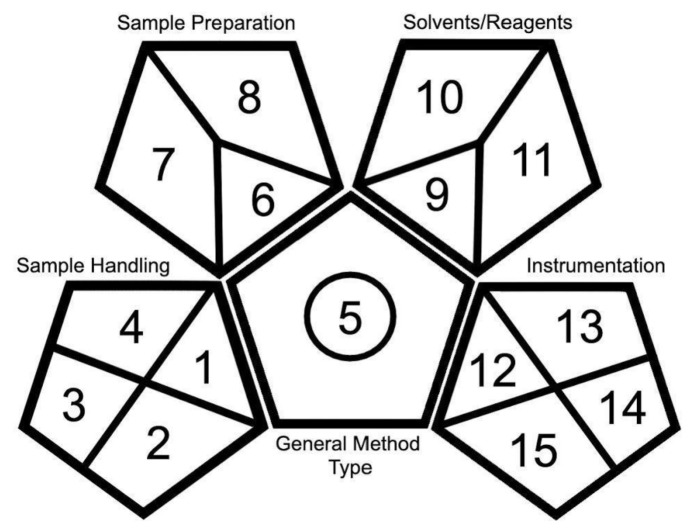
Layout and composition of the Green Analytical Procedure Index (GAPI) evaluation pictogram.

The greenness evaluation performed by this method is presented in the form of pictograms. The pictogram includes a pentagon in the center with four additional pentagons mirroring across each of the shape’s edges, sans the bottom edge (Figure 2). The central pentagon represents the general method type. The presence or absence of a circle in the central pentagon denotes if the method is quantitative or qualitative, respectively. The outer pentagons—beginning with the bottom-left and working around clockwise—represent the four main categories of assessment criteria: sample handling, sample preparation, required solvents and reagents, and instrumentation. Divisions made within these shapes denote individual lines of criteria. Evaluations of given criteria are equated to a color: components of the technique that correspond to the principles of GAC are given in green, components that somewhat correspond are given in yellow, and components that do not correspond with the principles are given in red [8]. Presenting the information in this way allows one to comprehend a great deal of information at a glance and allows for easy comparison.

Each of the analytical techniques compared in this paper are quantitative methods that require extraction. For this reason, all pictograms have a red central pentagon with a circle in the center. Similarly, each of these techniques used GC–MS instrumentation for analysis. As such, the instrumentation portion of the pictogram (bottom-right pentagon) is the same for each technique, with the exception of overall waste (tile 14).

## 3. SPME Methods Evaluation through GAPI Method

Four papers were chosen in which the authors developed new analytical methods based on SPME and compared their performance with other sample preparation techniques. For each paper, the GAPI method was used to evaluate the greenness of each technique and a pictogram was made to represent each evaluation. The pictograms were then used to compare the techniques and served as a visual aid in the discussion of their features. In this way, the greenness of SPME can be directly compared to that of other methods while simultaneously comparing the quality of data yielded from each technique. 

### 3.1. Interlaboratory Validation of a Thin-Film Microextraction Technique for Determination of Pesticides in Surface Water Samples 

**Figure 3:**
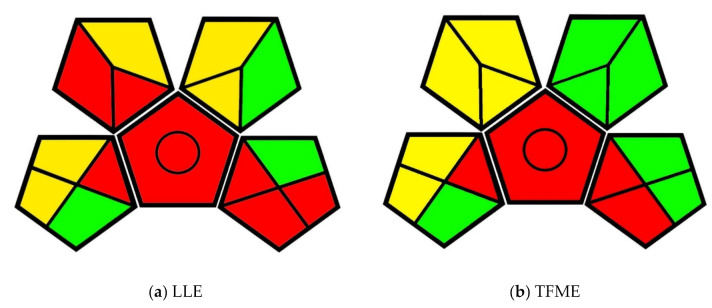
(**a**) GAPI evaluation pictogram for the liquid–liquid extraction (LLE) method; (**b**) GAPI evaluation pictogram for the thin-film microextraction (TFME) method in Piri-Moghadam et al., 2017 [32].

The first paper used for method greenness comparison is by Piri-Moghadam et al. [32]. In this paper, liquid–liquid extraction (LLE) and thin-film microextraction (TFME) techniques were used to assess the presence of pesticides in surface water. For this analysis, the LLE technique involved adjusting pH to acidic, neutral, and basic conditions, extracting 800 mL of each sample sequentially with dichloromethane, combining extracts, and analyzing an aliquot by GC–MS. The TFME technique involved adjusting the pH of 30 mL of nanopure water to 2.5 by phosphate buffer, adjusting the ionic strength adding 9 mg of sodium chloride, performing extraction at 900 rpm for 30 min, transferring the membranes to a liner compatible with a Thermal Desorption Unit (TDU), and introducing it to GC–MS by means of an autosampler. The GAPI evaluation pictograms for each of these techniques are seen below in Figure 3.

The collection, preservation, transport, and storage required for samples are the same for both analytical techniques, as seen in the bottom-right pentagon in both pictograms. However, the difference in greenness between these two techniques can be seen in the upper two pentagons. The volume of organic solvent and the degree of sample preparation required for the LLE technique are much greater than those of the TFME technique, as demonstrated in Figure 3. 

### 3.2. Fast and Robust Direct Immersion Solid-Phase Microextraction Coupled with Gas Chromatography–Time-Of-Flight Mass Spectrometry Method Employing a Matrix Compatible Fiber for Determination of Triazole Fungicides in Fruits 

The second comparison was done between the methods used by Souza-Silva et al. [33]. In this work, SPME and QuEChERS were used to analyze fungicides in fruits. For both methods of analysis, purchased fruit samples were stemmed, washed, crushed, and homogenized using a blender. The samples were then transferred to 200-mL glass flasks and stored at −30 °C. For this reason, the sample preparation portions of each pictogram exhibit the same greenness evaluation. 

**Figure 4:**
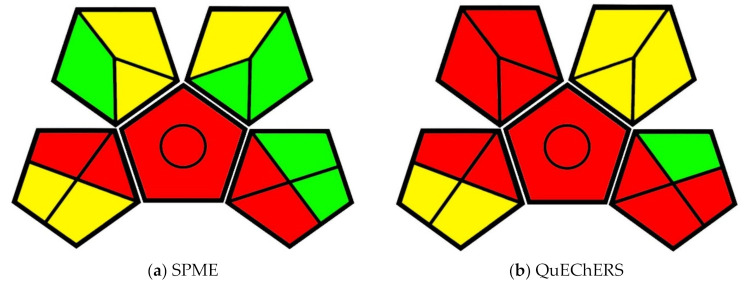
(**a**) GAPI evaluation pictogram for the solid-phase microextraction (SPME) method; (**b**) GAPI evaluation pictogram for the Quick Easy Cheap Effective Rugged Safe (QuEChERS) method from Souza-Silva et al., 2013 [33].

The SPME analysis involved transferring a 9 g aliquot of fruit pulp to a vial, adding standards and pre-incubating for at least 60 min prior to extraction. The extraction step then involved direct immersion of the fiber for 15 min while the sample was stirred at 500 rpm at 50 °C. The QuEChERS technique involved transferring 15 g of fruit pulp to a centrifuge tube and adding standards; the samples were left to homogenize for 60 min. Next, 15 mL acetonitrile (1% acetic acid) were mixed in, followed by the addition of 1.5 g sodium acetate and 6 g magnesium sulfate. The samples were then vortexed for 1 min and centrifuged at 3000 rpm for 2 min. 8 mL of extract, 400 mg of PSA (primary-secondary amine), and 1200 mg of magnesium sulfate were combined in a centrifuge tube and again vortexed for 1 min and centrifuged at 3000 rpm for 2 min. The final extract was analyzed by gas chromatography–time-of-flight mass spectrometry (GC–ToFMS). The GAPI evaluation for these methods can be seen in the pictograms below (Figure 4).

The difference in greenness between the sample preparation for these two methods is inherently different in that the SPME extraction is performed on a microscale, while QuEChERS extraction is performed on a macroscale. Furthermore, the QuEChERS method requires extraction solvents and a great deal of additional treatment, while SPME only requires incubation and agitation. These differences can be seen in the top-left pentagon of each pictogram. The lack of necessity for reagents and extraction solvents in SPME also means that the hazards associated with the procedure are greener as well. This is seen in the top-right portion of the pictograms. 

### 3.3. Comparison of Solid-Phase Microextraction to Solvent Extraction and QuEChERS for Quantitative Analysis of Veterinary Drug Residues in Chicken and Beef Matrices 

**Figure 5:**
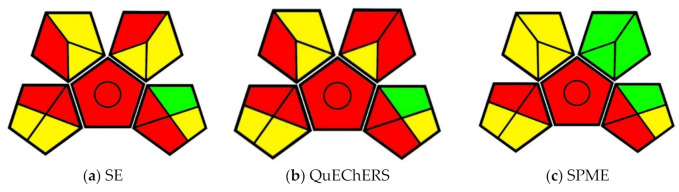
(**a**) GAPI evaluation pictogram for the solvent extraction (SE) method; (**b**) GAPI evaluation pictogram for the QuEChERS method; (**c**) GAPI evaluation pictogram for the solid-phase microextraction (SPME) method from Khaled et al., 2019.

Khaled et al. [34] developed an SPME analytical method to measure trace veterinary drug residues that make their way into consumer meat products, particularly chicken and beef. Food regulatory agencies have mandated that only specific levels of drug residues are acceptable in consumer meat products, thus establishing the need for analytical methods to detect the drugs in question. Two long-standing methods for measuring the drug residue levels in meat products are the solvent extraction (SE) and QuEChERS methods. Khaled et al. compared the two existing methods to their SPME method to determine effectiveness. The greenness of the methods was visually compared using the GAPI pictogram method (Figure 5).

All three methods under comparison make use of an LC–MS running with a mobile phase of water and MeCN with formic acid. While MeCN is a hazardous solvent, water is a green, renewable solvent. The formic acid is minimal (0.1% *v*/*v*), but also a safety concern. Each of the methods also makes use of energy-intensive preservation, as the tissue samples were homogenized using liquid nitrogen in a cryogenic grinder before being stored at −30 °C. 2.0 g of tissue samples were spiked with 200 µL of veterinary drug solutions before vortexing and 1 h of further agitation. The samples also had to be refrigerated after being prepared in this manner. Due to the high energy inputs, the methods earn red and yellow designations in the Storage and Preservation categories of the pictograms, respectively.

The differences in sustainability between the methods are visible beginning with the automated sample preparation station available for the SPME method. The automated system can also allow for the automatic injection of extracted samples, a capability that the SE and QuEChERS methods cannot utilize.

In the SE and QuEChERS methods, the samples were extracted using 10 mL of MeCN in water, whereas the SPME extractions were only carried out in 6 mL of water. For the use of MeCN, both the SE and QuEChERS methods earned red and yellow designations in the Health Hazard and Safety Hazard categories while the SPME method maintained its green designation. The SE method also required submitting 1.5 mL of the sample to five minutes of vortexing and three more minutes of centrifuging before automatic injection. The SPME method only required a minute of vortexing for its 1.5 mL sample, simplifying the number of steps to complete the method while also decreasing energy input and possible transfer waste. The QuEChERS method required the same volume, vortexing, and centrifuging as the SE method, as well as another round of vortexing (30 s) and centrifugation (three minutes) in a tube containing C18 sorbent, earning an orange distinction in the Additional Treatment category. This additional treatment also accrued more transfer waste and solvent use due to the extra steps. 300 µL of sample resulted from all methods and only 10 µL were needed for injection. The SPME method resulted in approximately 4.8 mL of waste, while the SE and QuEChERS methods produced 10.3 mL of waste. The use of extra steps could have resulted in more transfer waste in the QuEChERS and SE methods. Both the QuEChERS and SE methods earned a yellow designation in the Waste category. Although it produced less than half of the waste, the SPME method also earned an orange designation.

Overall, the SPME method is the greenest, and the QuEChERS method is marginally less green than the SE method. The difference as outlined by the GAPI evaluation is in the use of water instead of water and solvent extraction, as well as additional extraction steps that required energy input and created more waste. The SPME method was more efficient than the other methods because it required less agitation and waste to provide similar results.

### 3.4. Rapid and Concomitant Analysis of Pharmaceuticals in Treated Wastewater by Coated Blade Spray Mass Spectrometry 

Poole et al. [35] developed a coated blade spray thin-film solid-phase microextraction (CBS-TF-SPME) method to detect pharmaceutical contamination in water leaving wastewater treatment plants. The authors compared their method to a traditional SPE method outlined in Hao et al. [36]. One of the largest differences between the two methods was the overall scale: the SPE method used much larger quantities of material than the SPME method in the sample preparation and extraction phases.

**Figure 6:**
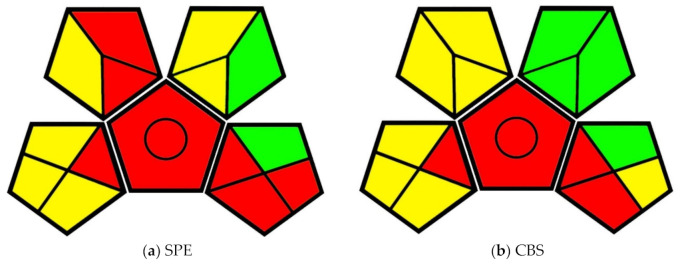
(**a**) GAPI evaluation pictogram for the solid-phase extraction (SPE) method; (**b**) GAPI evaluation pictogram for the coated blade spray thin-film solid-phase microextraction (CBS-TF-SPME) method from Poole et al., 2017.

In the SPE method, 400 mL of DI water were spiked with 1 mL of sample. The sample was then treated with 2 g of disodium EDTA and the method surrogate solution. This method also required additional treatment—pH adjustment using H_2_SO_4_ or NaOH. Already, the SPE method has earned a red designation in Waste, Extraction Scale, and Additional Treatment categories. The use of H_2_SO_4_ and NaOH also earned a yellow designation in the Health Hazard category (Figure 6).

The SPE extraction used Hydriphilic-Lipophilic Balance (HLB) sorbent, preconditioned with 5 mL of MeOH and 5 mL of water. After the extraction, the SPE cartridges were rinsed in 5 mL MeOH in water and dried under vacuum. The analytes were eluted with 5 mL more MeOH before being diluted in 100 µL of DI water for injection. The extraction procedure accrued another 20 mL of waste and added many more steps. In comparison, the CBS-TF-SPME method extraction used 9 mL of sample agitated in a shaker for ten minutes. The CBS devices were rinsed in water, following a desorption/ionization step using 15 μL of a 5:95 water:MeOH solution with minimal acetic and formic acid. The CBS method enabled direct introduction of the ionized analytes into a mass spectrometer without the need for chromatography, thus securing its place as the greener method by establishing a simpler procedure that minimizes waste, solvent use, and an overall number of required steps.

## 4. Conclusions

In this manuscript, we summarize how the principles of green analytical chemistry can be applied during the development and implementation of new analytical methods. In particular, we describe how sample preparation and extraction procedures can be dramatically improved by the use of sorbent-based microextraction technologies. The greenness of several analytical methods was assessed using the GAPI assessment tool, which provides a convenient visual representation of the compliance of analytical procedures to the principles of green analytical chemistry. The results of this assessment demonstrate that SPME can improve method greenness by offering a microscale extraction that uses little to no solvent, requires little preparation, and produces less waste than other types of extraction. As such, SPME provides indisputable benefits in analysis throughput and overall analytical performance.

## Figures and Tables

**Table 1:** 12 Principles of Green Chemistry and Green Analytical Chemistry.

Principle	Green Chemistry [1]	Green Analytical Chemistry [5]
1	Prevent waste production	Direct analysis should be used
2	Reactions are designed to be as efficient and atom economic as possible	Sample size and number should be minimized
3	Methods are modified to use fewer and less dangerous materials	When possible, analysis should take place in situ
4	Final products are as safe and sustainable as possible	Operations and analytical processes should be integrated to save resources
5	Methods are modified to use as few substances as possible, particularly solvents	When possible, analysis should be automated
6	Methods are energy efficient, conducted when possible at ambient temperature and pressure	Avoid derivatization
7	Materials should be sustainably or renewably sourced	Waste production should be minimized, proper waste management is essential
8	When possible, derivatization is avoided or decreased	When possible, the method should be optimized for use on multiple analytes to limit the amount of required testing
9	Selective catalytic reactions should be used in place of stoichiometric equivalents	Energy use should be minimal
10	Final products should be designed to degrade safely	Sustainable and renewable reagents are preferred
11	Waste production must be properly monitored	Safer reagents—less toxic, hazardous—are preferred
12	Procedures minimize danger and chemical accidents	Operator safety is paramount

**Table 2:** Different sample preparation techniques are compared by relative greenness factors. Colors indicate the level of compliance to the GAC principles, red: low, orange: medium, green: high.

	Organic Solvent Consumption	Energy Consumption	Time Consumption	Laboratory Waste	Reusability	Automation
Soxhlet extraction	High	High	High	High	No	No
Liquid–liquid	High	High	High	Medium	No	No
Wet and dry ashing	High	High	High	Low	No	No
Ultrasound-assisted extraction (UAE)	High	High	Low	Low	No	No
Pressurized solvent extraction (PSE)	Low	High	Low	Low	No	Yes
Microwave-assisted extraction (MAE)	None	High	Low	Low	No	Yes
Supercritical fluid extraction (SFE)	None	High	Medium	Low	Yes	No
QuEChERS	Medium	Low	Medium	High	No	Yes
Miniaturized QuEChERS	Low	Low	Low	Medium	No	Yes
Solid-phase extraction (SPE)	Medium	Low	Medium	High	Yes	Yes
Solid-phase microextraction (SPME)	Low	Low	Medium	Low	Yes	Yes
